# Chenodeoxycholic acid activates NLRP3 inflammasome and contributes to cholestatic liver fibrosis

**DOI:** 10.18632/oncotarget.13796

**Published:** 2016-12-04

**Authors:** Zizhen Gong, Jiefei Zhou, Shengnan Zhao, Chunyan Tian, Panliang Wang, Congfeng Xu, Yingwei Chen, Wei Cai, Jin Wu

**Affiliations:** ^1^ Department of Pediatric Surgery, Xinhua Hospital, Shanghai Jiaotong University School of Medicine, Shanghai, China; ^2^ Shanghai Institute for Pediatric Research, Shanghai Jiaotong University School of Medicine, Shanghai, China; ^3^ Shanghai Key Laboratory of Pediatric Gastroenterology and Nutrition, Shanghai, China; ^4^ State Key Laboratory of Proteomics, National Center for Proteomics Science (Beijing), Beijing Institute of Radiation Medicine, Beijing, China; ^5^ National Engineering Research Center for Protein Drugs, Beijing, China; ^6^ Shanghai Institute of Immunology, Institutes of Medical Sciences, Shanghai Jiaotong University School of Medicine, Shanghai, China

**Keywords:** bile acid, inflammasome, IL-1β, inflammation, liver fibrosis, Immunology and Microbiology Section, Immune response, Immunity

## Abstract

Accumulation of hydrophobic bile acids in the liver contributes to cholestatic liver injury. Inflammation induced by excessive bile acids is believed to play a crucial role, however, the mechanisms of bile acids triggered inflammatory response remain unclear. Recent studies have highlighted the effect of NLRP3 inflammasome in mediating liver inflammation and fibrosis. In this study, we for the first time showed that chenodeoxycholic acid (CDCA), the major hydrophobic primary bile acid involved in cholestatic liver injury, could dose-dependently induce NLRP3 inflammasome activation and secretion of pro-inflammatory cytokine-IL-1β in macrophages by promoting ROS production and K^+^ efflux. Mechanistically, CDCA triggered ROS formation in part through TGR5/EGFR downstream signaling, including protein kinase B, extracellular regulated protein kinases and c-Jun N-terminal kinase pathways. Meanwhile, CDCA also induced ATP release from macrophages which subsequently causes K^+^ efflux via P2X7 receptor. Furthermore, *in vivo* inhibition of NLRP3 inflammasome with caspase-1 inhibitor dramatically decreased mature IL-1β level of liver tissue and ameliorated liver fibrosis in bile duct ligation (BDL) mouse model. In conclusion, excessive CDCA may represent an endogenous danger signal to activate NLRP3 inflammasome and initiate liver inflammation during cholestasis. Our finding offers a mechanistic basis to ameliorate cholestatic liver fibrosis by targeting inflammasome activation.

## INTRODUCTION

Cholestasis is a pathological condition that occurs when bile outflow from the liver is impaired, which presents in multiple disorders including primary biliary cirrhosis, congenital biliary atresia, progressive familial intrahepatic cholestasis, drug hepatotoxicity and other forms of liver disease [1-3]. Persistent cholestasis leads to inflammation, hepatic stellate cells (HSCs) proliferation and eventually liver fibrosis, cirrhosis and even death. It is generally hypothesized that the accumulation of hydrophobic bile acids, which are considered to be highly toxic [4, 5], contributes to cholestatic liver injury. Numerous studies indicate that inflammation induced by toxic bile acid retention plays a integral role in the development of liver fibrosis, however, the molecular mechanism concerning the initiation of inflammatory response during cholestasis still remains unclear [6-9].

 Increasing evidence shows that the levels of multiple pro-inflammatory cytokines are strongly increased in the cholestatic liver tissue, including IL-1β, IL-6 and tumor necrosis factor α (TNF-α). As a key inflammatory mediator upstream of IL-6 and TNF-α signaling cascades, IL-1β is produced primarily by activated macrophages and has been demonstrated to be involved in diverse acute and chronic liver injury, including liver fibrosis [10-12]. IL-1β could activate HSCs and induce the production of matrix metalloproteinases (MMPs), promote the proliferation of fat storing cells and stimulate the synthesis of extracellular matrix (ECM), meanwhile, induce TGF-β synthesis and potentiate the injurious effects of TNF α [13], therefore facilitating liver fibrogenesis. Consistently, intervention with an IL-1 receptor antagonist was reported to protect rats from dimethylnitrosamine-induced liver fibrosis and markedly reduce alcohol-induced liver inflammation and fibrosis [12, 14]. In addition, IL-1β also has been observed to be associated with fibrosis in lung, kidney and other organs [15, 16].

 Recent studies have highlighted the critical role of inflammasome in the IL-1β production [17-19]. Inflammasome, including NLRP1, NLRP3, NLRC4, AIM2 family member, is a molecular platform that can be activated by pathogen-associated molecular patterns (PAMPs) as well as damage-associated molecular patterns (DAMPs) and trigger inflammatory response through promoting the maturation and secretion of highly pro-inflammatory cytokines, such as IL-1β. So far, NLRP3 inflammasome is the most fully characterized inflammasome and can be activated by a broad range of stimuli. Upon stimulation, NLRP3 assembles into a large cytoplasmic complexes by recruiting apoptosis associated speck-like protein (ASC) and caspase-1 (interleukin-1 converting enzyme, ICE), subsequent activation of caspase-1 leads to the cleavage of pro-IL-1β, which enables its maturation and secretion from the cells [20-23]. Thus, unlike most of the other cytokines, maturation and secretion of IL-1β undergoes two-step signal process. Firstly, IL-1β is synthesized as an inactive precursor form (pro-IL-1β), depending on the activation of NF-κB via Toll-like receptors which recognize PAMPs, such as LPS. Activation of NLRP3, is crucial in providing the secondary signal necessary for the production of mature IL-1β. Overproduction of IL-1β resulted from dysregulated NLRP3 inflammasome activation is proved to be associated with type 2 diabetes, rheumatoid arthritis, gout, atherosclerosis, and Alzheimer's disease, more importantly, emerging evidences suggest the pivotal role of NLRP3 inflammasome in mediating liver inflammation and fibrosis [24-26].

Dramatical increase of IL-1β during cholestasis indicates the presence of inflammasome activation. Liver is a major target organ of gut-derived bacteria and endotoxins, which could be promptly eradicated under the physiological conditions, whereas cholestasis is usually accompanied by increased translocation rate of bacteria and endotoxins (such as LPS) due to the intestinal microecological unbalance and mucosal barrier damage caused by bile acids deficiency in the gut, therefore translocated LPS may provide the first signal for the synthesis of pro-IL-1β. However, whether the accumulated toxic bile acid can serve as the danger signal to provoke the activation of inflammasome and subsequent production of mature IL-1β is unknown.

 Chenodeoxycholic acid (CDCA), the major hydrophobic primary bile acids, its concentration is strongly increased during cholestasis and higher than those of other toxic bile acids [27], thus is believed to be most implicated in the cholestatic liver injury. In this study, we focus our investigation on the role and mechanism of CDCA in the activation of NLRP3 inflammasome, and the effect of inflammasome inhibition on the liver fibrosis in the bile duct ligation (BDL) mouse model.

## RESULTS

### CDCA induces mature IL-1β secretion and caspase-1 activation in macrophages

**Figure 1:**
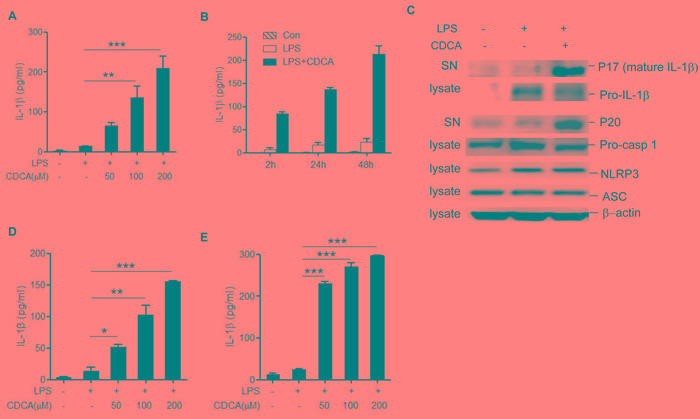
CDCA induces mature IL-1β secretion and caspase-1 activation in macrophages **A.** LPS-primed J774A.1 macrophages were incubated with various doses of CDCA for 24h. Secreted IL-1β was analyzed by ELISA. **B.** LPS-primed J774A.1 macrophages were treated with CDCA (100 μM) for different time courses. Secreted IL-1β was analyzed by ELISA. **C.** Immunoblot analysis of mature IL-1β (mIL-1β, 17kD), cleaved caspase-1 (p20, 20kD) in the culture supernatants (SN) and precursors of IL-1β (pro-IL-1β), caspase-1 (pro-caspase-1), NLRP3, ASC in lysates of LPS-primed J774A.1 macrophages stimulated with CDCA (100 μM) for 24h. β-actin was immunoblotted as a loading control. **D.**-**E.** ELISA analysis of IL-1β in supernatants from LPS-primed (D) BMDMs (E) kupffer cells treated with various doses of CDCA. *: *p* < 0.05; **: *p* < 0.01; ***: *p* < 0.001. Error bars indicate s.e.m. Representative data from at least 3 independent experiments giving similar results are shown.

 We hypothesized that CDCA may serve as the danger signal to activate macrophages and provoke the maturation of IL-1β, thus LPS-primed murine macrophage cell line J774A.1 was incubated with different dosage of CDCA. Results showed that CDCA exposure obviously induced IL-1β secretion in a dose and time-dependent manner (Figure 1A, 1B). The cleavage of procaspase-1, which produces active caspase-1 (p20), is essential to the IL-1β maturation [22, 23]. Consistently, western blotting analysis of cell culture supernatant confirmed caspase-1 activation and mature IL-1β (mIL-1β) release after CDCA incubation (Figure 1C). Meanwhile, CDCA was observed to exert similar effects on murine bone marrow derived macrophages (BMDMs) as well as liver kupffer cells (Figure 1D and 1E). Moreover, we treated macrophages with CDCA after LPS challenge, CDCA stimulation did not significantly increase the pro-IL-1β, NLRP3 and ASC level (Figure 1C), and CDCA alone had no obvious effect on mature IL-1β secretion or pro-IL-1β and NLRP3 expression (Supplementary Figure 1 and data not shown). Together these data demonstrate that CDCA significantly induces IL-1β maturation and release in macrophages, suggesting its effect on triggering inflammasome activation. Furthermore, we also determined the effect of TCA, the level of which is also obviously elevated during cholestasis, on the inflammasome activation.

Results showed that different from CDCA, TCA treatment did not induce obvious mature IL-1β production in LPS-primed macrophages (Supplementary Figure 2). The mechanisms responsible for the difference between bile acids on the induction of inflammasome activation need further investigation, while one possible explanation is that conjugated bile acids are less hydrophobic, which may be related to their incapability of inducing inflammasome activation.

### CDCA promotes the secretion of mature IL-1β in a NLRP3-dependent manner

**Figure 2:**
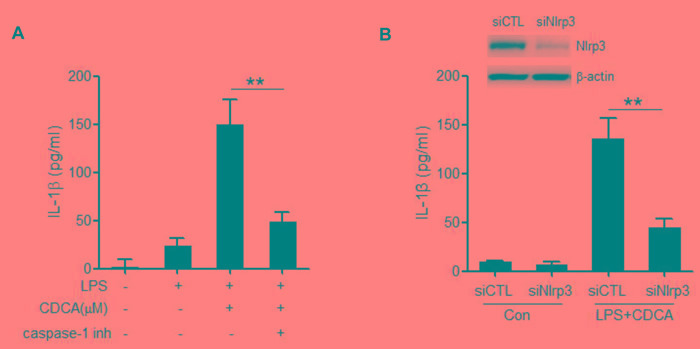
CDCA promotes the secretion of mature IL-1β in a NLRP3-dependent manner **A.** LPS-primed J774A.1 macrophages were treated with CDCA (100 μM) in the presence or absence of caspase-1 inhibitor (10 μM). IL-1β in supernatants was analyzed by ELISA. **B.** Control siRNA (siCTL) or Nlrp3 siRNA (siNlrp3) transfected J774A.1 macrophages were primed with LPS and then treated with CDCA (100 μM) for 24h. IL-1β in supernatants was analyzed by ELISA. Inset, Immunoblot analysis of NLRP3 expression in siCTL or siNlrp3 transfected cells. **: *p* < 0.01. Error bars indicate s.e.m. The data shown are representative of 3 individual experiments.

Caspase-1 inhibitor could significantly impair the IL-1β secretion induced by CDCA (Figure 2A), which further reveal that CDCA activates caspase-1 in macrophages, leading to IL-1β release. The NLRP3 inflammasome is the most fully characterized inflammasome and usually involved in caspase-1 activation induced by multiple danger signals. Here we found that treatment of LPS-primed J774A.1 macrophages with CDCA provoke substantial IL-1β secretion in macrophages transfected with control siRNA but not in those transfected with siRNA specific for Nlrp3 (Figure 2B). These data suggest that CDCA triggers the secretion of bioactive IL-1β mainly via the activation of NLRP3 inflammasome.

### CDCA-induced NLRP3 inflammasome activation depends on ROS production and potassium efflux

**Figure 3:**
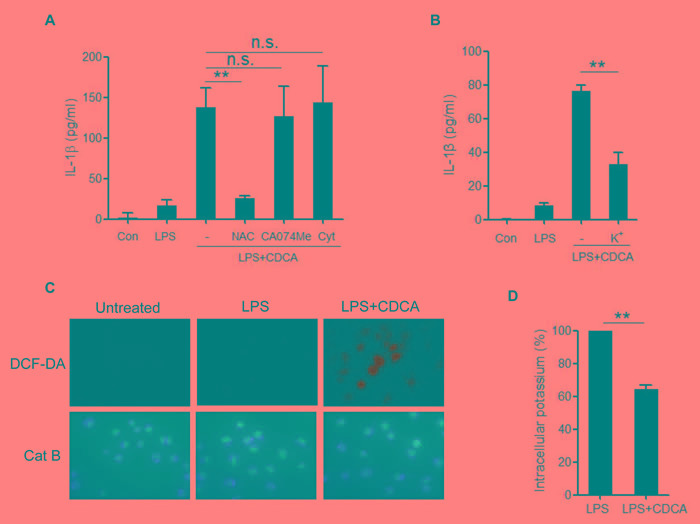
CDCA-induced NLRP3 inflammasome activation requires ROS production and potassium efflux **A.** LPS-primed J774A.1 macrophages were treated with CDCA in the presence or absence of NAC (10 mM), CA-074Me (50 μM), or Cytochalasin D (2.5 μM) for 24h. IL-1β in supernatants was measured by ELISA. **B.** LPS-primed J774A.1 macrophages were treated with CDCA in the presence or absence of KCl (140 mM) for 2h. IL-1β in supernatants was measured by ELISA. **C.** LPS-primed J774A.1 macrophages were treated with or without CDCA. The cells were incubated with DCF-DA probe (green) or cathepsin B (Cat B) substrate z-Arg-Arg-cresyl violet, which emits red signal upon cleavage by cathepsin B, followed by Hoechst staining (blue). **D.** LPS-primed J774A.1 macrophages were treated with or without CDCA, and intracellular potassium level was measured by ICP-OES. **: *p* < 0.01; n.s.: no statistically significant difference (*p* > 0.05). Error bars indicate s.e.m. The data shown are from 3 independent experiments.

NLRP3 inflammasome activation may occur through crystal phagocytosis, reactive oxygen species (ROS) formation, potassium (K+) efflux and cathepsin B leakage [28-31]. To identify the mechanisms through which NLRP3 inflammasome is activated by CDCA, we observed the effect of selective inhibitors on IL-1β secretion in CDCA-treated macrophages. Cytochalasin D, a phagocytosis-blocking agent and CA-074Me, a cathepsin B inhibitor had no obvious effect, whereas the antioxidant N-acetyl-L-cysteine (NAC) could significantly attenuate CDCA-induced IL-1β secretion (Figure 3A). Furthermore, prevention of K+ efflux by using high concentrations of KCl in the culture medium also suppressed CDCA-induced IL-1β secretion (Figure 3B), which indicates the involvement of K+ efflux in the CDCA-induced inflammasome activation. By using DCF-DA, an indicator of intracellular ROS generation and Cathepsin B fluorogenic substrate (z-Arg-Arg cresyl violet), we confirmed ROS formation in CDCA-treated macrophages and excluded the presence of cathepsin B leakage (Figure 3C). Meanwhile, we indeed observed the profound decrease of intracellular potassium concentration in CDCA-treated cells (Figure 3D). Taken together, these data suggest that CDCA induces NLRP3 inflammasome activation mainly through promoting ROS generation and K+ efflux.

### TGR5-transactivated EGFR signaling contributes to the CDCA-induced ROS production

**Figure 4:**
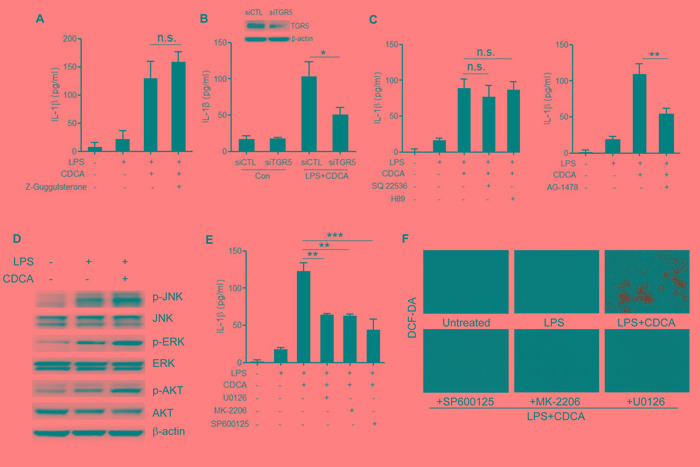
TGR5/EGFR signaling contributes to the CDCA-induced ROS production and IL-1β secretion **A.** LPS-primed J774A.1 macrophages were treated with CDCA in the presence or absence of Z-Guggulsterone (20 μM). IL-1β in supernatants was analyzed by ELISA. **B.** Control siRNA (siCTL) or TGR5 siRNA (siTGR5) transfected J774A.1 macrophages were primed with LPS and then treated with CDCA for 24h. IL-1β in supernatants was analyzed by ELISA. Inset, Immunoblot analysis of TGR5 expression in siCTL or siTGR5 transfected cells. **C.** LPS-primed J774A.1 macrophages were treated with CDCA in the presence or absence of SQ22536 (400 μM), H89 (10 μM) or AG-1478 (30 μM). IL-1β in supernatants was analyzed by ELISA. **D.** Immunoblot analysis of phospho-JNK, total JNK, phospho-ERK, total ERK, phospho-AKT and total AKT of LPS-primed J774A.1 macrophages treated with or without CDCA. β-actin was immunoblotted as a loading control. (E-F) LPS-primed J774A.1 macrophages were treated with CDCA in the presence or absence of U0126 (10 μM), MK-2206 (10 μM) or sp600125 (25 μM). **E.** IL-1β in supernatants was analyzed by ELISA, **F.** Cells were incubated with DCF-DA probe for 1 h. Fluorescence images was used to exhibit the ROS formation. *: *p* < 0.05; **: *p* < 0.01; ***: *p* < 0.001; n.s.: no statistically significant difference (*p* > 0.05). Error bars indicate s.e.m. The data shown are representative of 3 individual experiments yielding similar results.

We sought to better understand the molecular mechanism underlying the activation of NLRP3 inflammasome by CDCA. In addition to their lipid-emulsifying properties, bile acids also serve as signaling molecules acting through two major receptors, the nuclear receptor farnesoid X-receptor (FXR) and membrane receptor TGR5 [32], both of which are found to be expressed in macrophages. Pre-treatment of LPS-primed macrophages with FXR antagonist (z-Guggulsterone) had no obvious effect on IL-1β secretion induced by CDCA (Figure 4A), while knockdown the expression of TGR5 significantly inhibited IL-1β release (Figure 4B), suggesting the role of TGR5 in mediating CDCA-induced inflammasome activation. As a G-protein-coupled receptor, TGR5 is reported to couple to Gs, resulting in the activation of adenylate cyclase (AC) and subsequent protein kinase A (PKA) downstream signaling. Here we found that both AC-inhibitor (SQ22536) and PKA inhibitor (H89) could not affect IL-1β secretion induced by CDCA, indicating the involvement of AC-independent signaling pathways (Figure 4C). Recent studies have linked TGR5 to epidermal growth factor receptor (EGFR). Bile acids have been reported to transactivate EGFR in cholangiocytes as well as colon cancer cells [33, 34]. More importantly, EGFR have been demonstrated to mediate high glucose-induced cardiac oxidative stress and be responsible for ROS production via EGFR-AKT/ERK signaling pathway [35, 36]. Based on our findings that ROS production contributes to CDCA-induced inflammasome activation, we pre-treated LPS-primed macrophages with EGFR inhibitor (AG-1478) and observed that EGFR inhibition could strongly reduce IL-1β secretion induced by CDCA (Figure 4C). Meanwhile, CDCA stimulation could dramatically up-regulate the phosphorylation of ERK1/2, AKT and JNK, three major signaling cascades downstream of EGFR (Figure 4D). Indeed, LPS-primed macrophages pre-treated with the ERK1/2 inhibitor (U0126), AKT inhibitor (MK-2206) or JNK inhibitor (SP600125) produced much less IL-1β in response to CDCA (Figure 4E) and pre-incubation with ERK, AKT or JNK inhibitor could substantially reduce the ROS production in CDCA-activated macrophages (Figure 4F). These results suggest that the induction effect of CDCA on NLRP3 inflammasome activation may be at least partially mediated through TGR5/EGFR signaling pathway which contributes to ROS production.

### CDCA induces ATP release from macrophages

**Figure 5:**
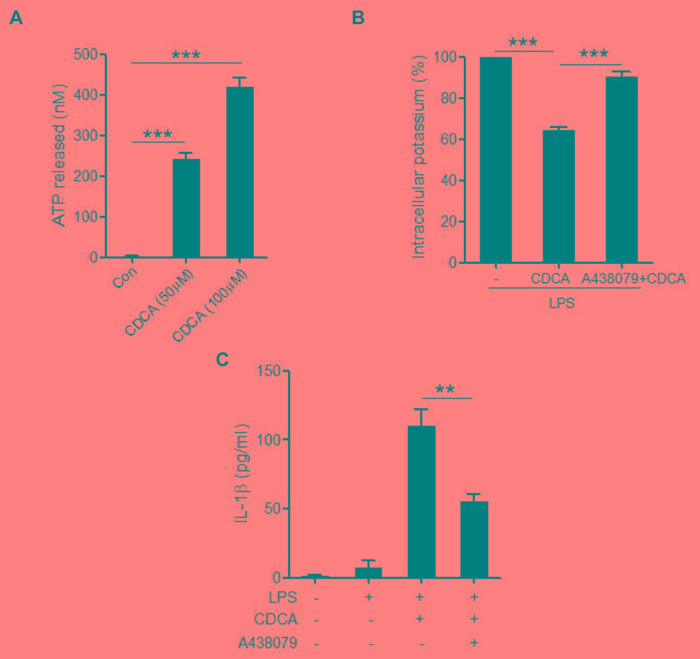
CDCA induces ATP release from macrophages **A.** J774A.1 macrophages were incubated with various doses of CDCA for 1h. ATP level in culture supernatants was determined by luciferase-luciferin reaction. **B.** LPS-primed J774A.1 macrophages were treated with CDCA in the presence or absence of A438079 (100 μM), and intracellular potassium level was measured by ICP-OES. **C.** LPS-primed J774A.1 macrophages were treated with CDCA in the presence or absence of A438079 (100 μM). IL-1β in supernatants was analyzed by ELISA. **: *p* < 0.01; ***: *p* < 0.001. Error bars indicate s.e.m. The data shown are from 3 independent experiments.

CDCA was proved to cause concentration-dependent ATP release from exocrine pancreatic cells [37]. As a potent activator of the NLRP3 inflammasome, ATP can trigger rapid K+ efflux from the cells through activation of P2X7 receptor, an ATP-gated ion channel. Based on our findings that K+ efflux is also involved in the CDCA-induced NLRP3 inflammasome activation, we attempted to determine whether the decrease of intracellular K+ level is attributed to ATP release from macrophages in response to CDCA. Using luciferase assay, we found that CDCA could dose-dependently induce significant amount of ATP release from macrophages (Figure 5A), and P2X7 receptor antagonist (A437980) almost completely reversed the reduction of intracellular K+ concentration caused by CDCA treatment (Figure 5B). Consistently, pre-treated LPS-primed J774A.1 macrophages with A437980 dramatically decrease IL-1β secretion induced by CDCA (Figure 5C). Thus, these data suggest that ATP release from macrophages upon CDCA stimulation contributes to K+ efflux and subsequent NLRP3 inflammmasome activation.

### Caspase-1 antagonist belnacasan attenuates liver inflammation and fibrosis in BDL mouse model

**Figure 6:**
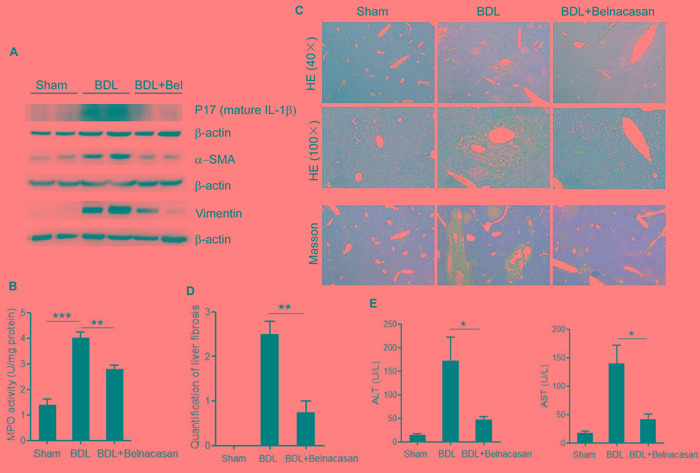
Caspase-1 antagonist attenuates liver inflammation and fibrosis in BDL mouse model **A.** Immunoblot analysis of mature IL-1β (17kD), α-SMA and Vimentin in liver homogenates of control, BDL and BDL plus Caspase-1 antagonist-treated mice. **B.** Liver MPO activity **C.** HE and Masson staining **D.** Liver fibrosis quantification **E. **Serum ALT and AST levels of control, BDL and BDL plus Caspase-1 antagonist-treated mice. *: *p* < 0.05; **: *p* < 0.01; ***: *p* < 0.001. Error bars indicate s.e.m. Representative data from 3 independent experiments are shown.

To investigate the role of NLRP3 infalmmasome activation and IL-1β release in the liver injury under cholestasis condition, caspase-1 inhibitor (belnacasan) was administrated to the mice underwent bile duct ligation (BDL). Mature IL-1β (p17) as well as MPO activity in liver homogenate increased dramatically compared to the sham-operated control. Administration of belnacasan exhibited profound improvement in BDL-induced liver inflammation (Figure 6A, 6B, 6C). Meanwhile, liver fibrosis, as assessed by smooth muscle actin (SMA), vimentin and Masson staining was significantly ameliorated by belnacasan treatment (Figure 6A, 6C, 6D). Liver injury was also substantially alleviated as evidenced by decreased ALT and AST levels (Figure 6E).Therefore, these results indicate that the NLRP3 inflammasome activation and IL-1β maturation plays an important role in cholestatic liver damage.

## DISCUSSION

**Figure 7:**
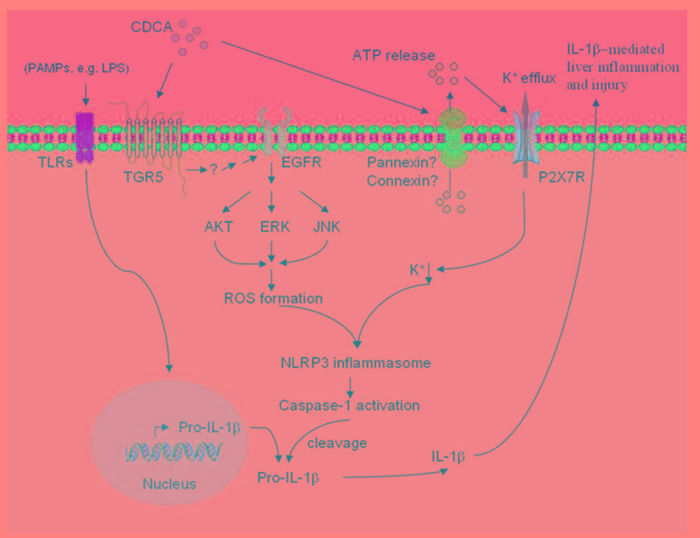
Proposed model of CDCA-induced NLRP3 inflammasome activation and liver injury CDCA promotes ROS formation in macrophages through TGR5/EGFR downstream signaling (AKT, ERK, JNK pathways), meanwhile, induces ATP release which subsequently triggers K^+^ efflux mainly via P2X7 receptor. ROS production and K^+^ efflux, as two of the major factors responsible for NLRP3 inflammsome activation, lead to the activation of caspase-1 and the maturation and secretion of pro-inflammatory cytokine IL-1β, thereby initiating liver inflammation and further injury.

Inflammation induced by accumulated toxic bile acids in the liver contributes to cholestatic liver fibrosis. However, the mechanisms by which toxic bile acids trigger inflammatory response remain elusive. The exploration of the initiating events of the inflammatory response may be beneficial to discover novel therapeutic strategies against cholestatic liver injury and fibrosis. In the current study, we for the first time proved that CDCA, the major toxic bile acid involved in cholestatic liver injury, could dose-dependently induce NLRP3 inflammasome activation and subsequent secretion of potent pro-inflammatory cytokine-IL-1β in murine J774A.1 macrophages, bone marrow derived macrophages as well as kupffer cells. Meanwhile, mature IL-1β levels in liver of BDL mouse model also enormously increased. IL-1β was reported to promote fibrogenesis and as an important inflammatory mediator, it can also recruit other inflammatory cells, especially neutrophils, which are actively implicated in tissue inflammation and exacerbate liver damage [6]. Consistently, our *in vivo* studies showed that inhibition of IL-1β level with caspase-1 inhibitor dramatically reduced MPO activity in the liver and ameliorated liver fibrosis in BDL mouse model, suggesting an important role of IL-1β in triggering tissue inflammation and fibrosis during cholestasis. Thus, our data provide a new mechanism that excessive toxic bile acids initiate liver inflammation at least in part through activating NLRP3 inflammasome (Figure 7).

Our *in vitro* studies demonstrated that phagocytic uptake was not required for CDCA-induced IL-1β secretion in macrophages, which is different from the mode of NLRP3 activation by various crystals, indicating the involvement of receptor-mediated pathways. The following experiments confirmed that CDCA activated NLRP3 inflammasome and induced IL-1β release partially through TGR5. TGR5 belongs to G protein-coupled receptor family and is widely expressed in cells and tissues, including gall bladder, brown adipose tissue, intestinal and endocrine cells, cholangiocytes, but not hepatocytes, thus mediates diverse biological effects [38]. In addition, TGR5 was also observed to exhibit immunomodulatory effects through its abundant expression on monocytes and macrophages. However, the role of TGR5 activation on inflammation has been controversial. In contrast to our results, some studies have shown that CDCA exerts an inhibitory effect on inflammatory cytokines production in LPS-stimulated macrophages mainly through TGR5-AC-cAMP dependent pathways [39]. Based on previous reports, bioactive IL-1β production requires priming signal, which increases pro-IL-1β and NLRP3 level, and then followed by activation signal (second signal) which induces NLRP3 inflammasome assembly and activation. Indeed, we observed mature IL-1β release from LPS-primed macrophages in response to CDCA, therefore, one possible explanation for the discrepancy is that we prime macrophages with LPS first and then use CDCA as second signal activating the inflammasome, whereas different strategy was applied in the others studies [39, 40]. Meanwhile, Our results showed that transactivation of EGFR, which is an alternative pathway independent of AC-cAMP, leads to inflammasome activation and IL-1β release, suggesting that TGR5 may regulate inflammatory response through different mechanisms. Considering that TGR5 is currently regarded as a potential target for drug development in metabolic and inflammatory diseases [41], our findings indicate that different effects of TGR5 agonists on cytokine production should be checked in the future pharmaceutical studies.

The activation of NLRP3 inflammasome by CDCA involves the up-regulation of EGFR-ERK/AKT/JNK signaling. Many GPCR agonists were observed to mediate EGFR transactivation and the linkage between TGR5 and EGFR has already been established in numerous studies [42, 43]. However, the mechanisms responsible for bile acid-mediated EGFR transactivation in macrophages have not been well defined. EGFR transactivation usually includes matrix metalloproteinases (MMPs) activation and subsequent cleavage of EGFR ligands, including epidermal growth factor (EGF), transforming growth factor-α (TGF-α), or heparin-binding EGF-like growth factor (HB-EGF), resulting in their release from cell membrane and binding-activation of EGFR [44, 45]. We indeed found that disruption with the MMP inhibitor attenuated CDCA-induced IL-1β production (data not shown), but the mechanisms that CDCA activates MMP via TGR5 and which EGFR ligands are involved still remain to be investigated. NLRP3 inflammasome activation by CDCA requires ROS formation, as evidenced by significantly reduced IL-1β production after NAC treatment. Importantly, several studies have described the induction of ROS by EGFR activation [35, 46]. Sh-RNA silencing of EGFR significantly inhibited high glucose-induced ROS generation in cardiomyocytes, and EGFR inhibition was observed to alleviate renal oxidative stress and fibrosis in the obesity-related nephropathy. In agreement with these reports, here we demonstrated that EGFR signaling pathways are also implicated in CDCA-induced ROS production in macrophages.

The present study shows that the role of CDCA on inflammasome activation is also attributed to the induction of ATP release from macrophages, which could trigger K+ efflux through purinergic signalling. The ATP release upon CDCA stimulation was formerly described for exocrine pancreatic cells, which could further regulate epithelial functions of pancreas [37]. Mechanistically, ATP release is usually mediated through pannexin, connexin, or other ion channels [47], in addition, CDCA can also incorporate into cell membranes, which may increase membrane fluidity and facilitate ATP release, the detailed process responsible for CDCA-induced ATP release from macrophage need further investigation. Moreover, we found that P2X7 receptor is involved in CDCA-induces K+ efflux, which is consistent with previous reports showing that ATP can bind to P2X7 receptor and then open the P2X7-associated ion channel pore, thereby increasing K+ conductance. Here we can not exclude the involvement of other K+ channels, although inhibition of P2X7 receptor almost restored the intracellular K+ concentration reduced by CDCA.

Taken together, our findings provide direct evidence that excessive CDCA can serve as endogenous danger signal to activate NLRP3 inflammasome by TGR5/EGFR signaling and promoting ATP release, therefore, identifying a new mechanism by which inflammation is initiated during cholestasis. Combined with others' reports showing that NLRP3 inflammasome activation plays a central role in the induction of inflammation and fibrosis in various liver pathologies such as nonalcoholic steatohepatitis (NASH) and alcoholic steatohepatitis (ASH) [26], and IL-1 participates in both the early fibrogenesis and the later maintenance of fibrosis in liver [10], these data suggest that targeting inflammasome activation related signaling pathways may represent an appealing strategy to ameliorate cholestatic liver fibrosis.

## MATERIALS AND METHODS

### Reagents

Lipopolysaccharide (LPS), Chenodeoxycholic acid (CDCA), CA-074 Me, Tyrphostin AG 1478, A438079 and N-acetyl-L-cysteine (NAC) were purchased from Sigma-Aldrich (St. Louis, MO). SQ22536 was obtained from Abcam (Cambridge, MA). Cytochalasin D and Z-Guggulsterone were purchased from Santa cruz Biotechnology (Santa cruz, CA). VX-765 (belnacasan) and MK-2206 were purchased from Selleck ( Houston, TX). SP600125 and U0126 were obtained from Cell Signaling Technologies (Beverly, MA). RPMI 1640, DMEM and antibiotics were obtained from Invitrogen (Carlsbad, CA). 2’, 7’-dichlorofluorescein diacetate (DCF-DA) were from Invitrogen/Molecular Probes. ELISA Kits were purchased from eBioscience (San Diego, CA).

### Mice

6- to 8-week-old C57BL/6 mice were purchased from Experimental Animal Center of the Chinese Academy of Sciences (Shanghai, China) and maintained in a specific pathogen free (SPF) facility. All experiments were performed according to the Guide for the Care and Use of Medical Laboratory Animals issued by the Ministry of Health of China and approved by the Shanghai Laboratory Animal Care and Use Committee.

### Cells

The murine macrophage cell line J774A.1 was obtained from Type Culture Collection of the Institutes of Biomedical Sciences, Fudan University (Shanghai, China). Cells were cultured in DMEM medium (Invitrogen) supplemented with 10% fetal bovine serum (Gibco) and 1% penicillin/streptomycin (Invitrogen) at 37°C with 5% CO2. Bone marrow derived macrophages (BMDMs) were obtained and cultured as described elsewhere [48]. Mouse Kupffer cells were isolated from C57BL/6 mice by collagenase perfusion and centrifugation on a Percoll density gradient (GE Healthcare, Pittsburgh, PA, USA).

### *in vitro* CDCA treatment

J774A.1 cells, BMDM or Kupffer cells were primed with 1μg/ml LPS for 5 h before stimulation with CDCA at different concentrations, then supernatants were harvested at indicated time points and the IL-1β level was determined by Mouse IL-1 beta (IL-1β) ELISA Kit (eBioscience) according to the manufacturer's instructions.

### Western blot

Cells were lysed by protein lysis buffer (Sigma) containing protease and phosphatase inhibitors (Theromo), and the supernatant was concentrated by acetone precipitation. Cell lysates (50 μg) or concentrated supernatant proteins were resolved by SDS-PAGE, transferred to PVDF membranes and probed with antibodies against IL-1β (Cell Signaling Technologies), Caspase-1 (Santa cruz), NLRP3 (R&D), ASC (Santa cruz), TGR5 (Abcam), and β-actin (sigma). For the detection of phosphorylated ERK, JNK and AKT, membranes were probed with specific antibodies against phospho-ERK (p-ERK), p-JNK or p-AKT (Cell Signaling, Beverly, MA, USA), and then re-probed with antibodies against total ERK, JNK and AKT after incubation with a stripping buffer (Thermo Fisher Scientific, Waltham, MA, USA). Reactive signals were detected by enhanced chemiluminescence (Thermo Fisher Scientific, Waltham, MA, USA) and ChemiDoc™ XRS+ System (Bio-Rad).

### Reactive oxygen species (ROS) measurement

LPS primed J774A.1 cells were treated with or without CDCA (100 μM, 24h) and ROS production was measured by using 2’, 7’-dichlorofluorescein diacetate (DCF-DA, Invitrogen) probes according to the manufacturer's instructions. Briefly, cells were incubated with DCF-DA (15 μM) for 1 h at 37°C after CDCA stimulation. Fluorescence was visualized directly under a fluorescence microscope. To evaluate the effect of different signal pathways on the ROS production induced by CDCA, U0126 (10 μM), MK-2206 (10 μM) or sp600125 (25 μM) was added to the culture medium 30 minutes ahead of CDCA treatment and then ROS production was measured.

### Cathepsin B imaging

LPS primed J774A.1 cells were incubated with or without CDCA (100 μM, 24h), then cells were stained with Cathepsin B fluorogenic substrate (Neuromics) for 1 hour, followed by Hoechst staining. Fluorogenic signals were captured by inverted fluorescence microscope (Leica).

### ICP-OES assay

LPS primed J774A.1 cells (1×107) were treated with or without CDCA (100 μM, 24h) , in some experiments, P2X7 inhibitor A438079 (100 μM) was added 30 minutes before CDCA treatment. The cells were lysed in ultra pure nitric acid before microwave digestion and then diluted to 5% HNO3. Intracellular K+ was analyzed by using Perkin Elmer Optima 8000 ICP-OES Spectrometer. External K+ calibration was performed between 0 and 10 ppm.

### Transfection of small interfering RNA oligonucleotides

J774A.1 macrophages cultured in 6-well plates were transfected with NLRP3, TGR5 small interfering RNA or scrambled siRNA using TransIT-Jurkat (Mirus Bio, Madison, WI), followed by LPS stimulation and CDCA treatment (100 μM, 24h). IL-1β in supernatant was determined by ELISA. RNA oligonucleotides sequences were as follows: NLRP3, forward 5’- GGC GAG ACC UCU GGG AAA ATT-3’ and reverse 5’-UUU UCC CAG AGG UCU CGC CTT-3’; TGR5, forward 5’- CUG GAA CUC UGU UAU CGC UTT-3’ and reverse 5’-AGC GAU AAC AGA GUU CCA GTT-3’.

### ATP release

ATP level in culture supernatant was determined by ATP Assay System (Promega). Briefly, LPS primed J774A.1 cells were treated with or without CDCA for 1 hour in DMEM medium containing 0.1% FBS. Supernatants were collected and centrifuged for the measurement of ATP using luciferase-luciferin reaction.

### Bile duct ligation and belnacasan treatment

Mice were subjected to Bile Duct Ligation (BDL) as described previously and then were randomly divided into 2 groups after ligation (n=8 in each group). The vehicle group was treated with Cremophor EL, while the caspase-1 inhibitor group was given 50 mg/kg/day belnacasan (Selleck, Houston, TX) intraperitoneally everyday (dissolved in 25% Cremophor EL solution). Mice that did not undergo ligation of bile duct served as sham-operated controls. After 21 days, mice were sacrificed and liver tissue sections were prepared. Liver inflammation was evaluated by hematoxylin eosin (HE) staining and myeloperoxidase (MPO) activity measurement. Liver fibrosis was assessed with Masson staining and quantified by pathologist. 50 μg whole-liver lysate was used for immunoblot analysis of IL-1β, α-smooth muscle actin (α-SMA) and Vimentin. Meanwhile, blood was also collected and analyzed for ALT and AST level.

### Statistics

All results were presented as mean ± standard error of the mean (s.e.m.). Data were analyzed by 2-tailed Student's t-test or one-way analysis of variance (ANOVA). Differences were considered significant at p<0.05.

## SUPPLEMENTARY MATERIALS FIGURES AND TABLES


