# Supplementary material for: Chenodeoxycholic acid activates NLRP3 inflammasome and contributes to cholestatic liver fibrosis
Source: Oncotarget. 2016 Dec 4;7(51):83951–63. doi: 10.18632/oncotarget.13796 (PMC5356637; doi:10.18632/oncotarget.13796)
Supplement: Supplementary file 1 [file oncotarget-07-83951-s001.pdf]

Chenodeoxycholic acid activates NLRP3 inflammasome and contributes to cholestatic liver fibrosis

Supplementary Material

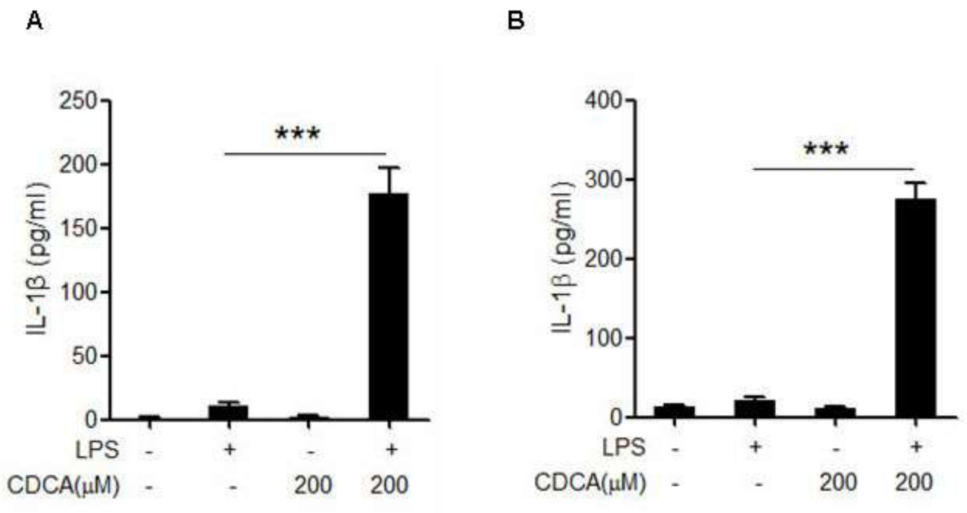

Supplementary Figure 1: CDCA induces the production of mature IL-1β in LPS-primed macrophages. (A) J774A.1 macrophages were exposed to CDCA (200μM) for 24h with or without LPS pre-treatment. Secreted IL-1β was then analyzed by ELISA. (B) Kupffer cells were exposed to CDCA (200μM) for 24h with or without LPS pre-treatment. Secreted IL-1β was analyzed by ELISA. ***: $p < 0.001$.

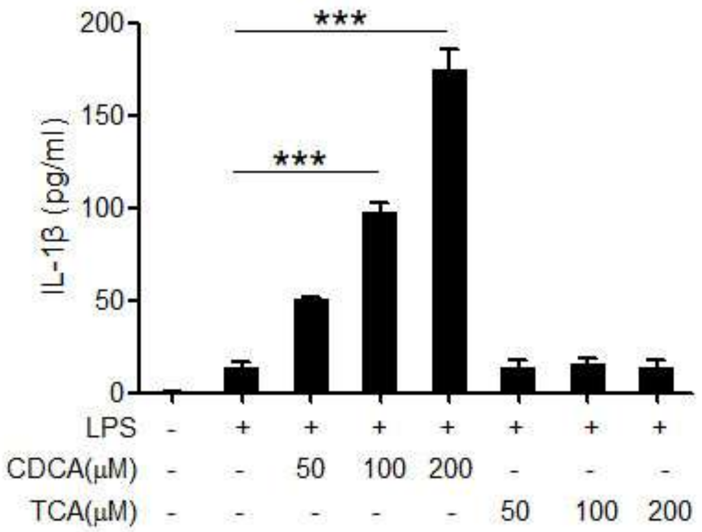

Supplementary Figure 2: The effect of TCA on the induction of IL-1β secretion. LPS-primed J774A.1 macrophages were incubated with various doses of CDCA or TCA for 24h. Secreted IL-1β was analyzed by ELISA. ***: $p < 0.001$.
